# High Performance Aqueous Li-Ion Flow Capacitor Realized Through Microstructure Design of Suspension Electrode

**DOI:** 10.3389/fchem.2021.673179

**Published:** 2021-04-20

**Authors:** Defu Cao, Xiaojie Bai, Junhui Wang, Hao Liu, Libing Liao

**Affiliations:** ^1^Beijing Key Laboratory of Materials Utilization of Nonmetallic Minerals and Solid Wastes, School of Materials Science and Technology, China University of Geosciences, Beijing, China; ^2^School of Science, China University of Geosciences, Beijing, China

**Keywords:** spinel LiMn_2_O_4_, polyaniline, electronic conductivity, viscosity, microstructure, large-scale energy storage

## Abstract

Suspension electrode is the core of flowable electrochemical energy storage systems, which are considered suitable for large-scale energy storage. Nevertheless, obtaining suspension electrodes with both low viscosity and high conductivity is still a big challenge. In present work, spinel LiMn_2_O_4_ was chosen as an example to make suspension with low viscosity and high conductivity through microstructure morphology control of solid particles and the contact mode between active materials and conductive additives in suspension electrode. By coating a thin layer of polyaniline on the surface of spherical spinel LiMn_2_O_4_, the resulting suspension showed much higher electronic conductivity (about 10 times) and lower viscosity (about 4.5 times) as compared to irregular and bare spinel LiMn_2_O_4_-based suspension counterpart. As a result, the Li-ion flow capacitor based on LiMn_2_O_4_ and activated carbon suspensions exhibited a record energy density of 27.4 W h L^−1^ at a power density of 22.5 W L^−1^ under static condition to date, and can be smoothly work under an intermittent-flow mode. The strategy reported in this work is an effective way for obtaining suspension electrodes with low viscosity and high electronic conductivity simultaneously. It can not only be used in the flow capacitors, but also can be extended to other flowable electrochemical energy storage systems.

## Introduction

With the rapid growth of installed capacity of clean energy such as solar energy and wind energy, the research and development of technologies and devices suitable for large-scale energy storage has become increasingly important (Chu and Majumdar, 2012; Carbajales-Dale et al., 2014). Electrochemical storage devices based on flowable suspension-type (i.e., semi-solid) electrodes have gained much attraction in the last decade (Campos et al., 2013; Zhang C. et al., 2014; Zhang X. et al., 2014; Hatzell et al., 2015; Madec et al., 2015; Liu and Zhao, 2016; Wang et al., 2016; Hou et al., 2017; Liu et al., 2017; Hunt et al., 2019; Wu et al., 2020). Similar to redox flow batteries, they usually consists of separated external reservoirs for active materials storage and a power reactor for electrochemical reaction to take place, which can offer good scalability and flexibility in application for many energy storage fields (Liu et al., 2017).

Suspension electrode is the core of flowable electrochemical energy storage systems. In general, the suspension electrode is a mixture of solid active materials, conductive additives, and liquid electrolyte. An ideal suspension electrode should own high conductivity for electron fast transfer and high power density during the charging/discharging processes, and low viscosity for less energy loss in pumping process. Nevertheless, obtaining suspension electrodes with both low viscosity and high conductivity is still a big challenge. On the one hand, suspension electrodes belong to non-Newtonian fluid, and their viscosity is mainly determined by the internal interaction force between solid particles (especially van der Waals force), which usually relates to the shape, size, particle size distribution, and content of solid particles (Madec et al., 2015). On the other hand, in order to improve the electrical conductivity of the suspension electrode, a large amount of conductive additives need to be added. Carbon nanoparticles are often used as conductive additives. Nevertheless, the viscosity can greatly increase after adding carbon additives because of its high specific surface area and thus high interaction between solid particles (Youssry et al., 2013). Therefore, to some extent, realizing low viscosity and high conductivity is contradictory for suspension electrodes.

**Figure 1:**
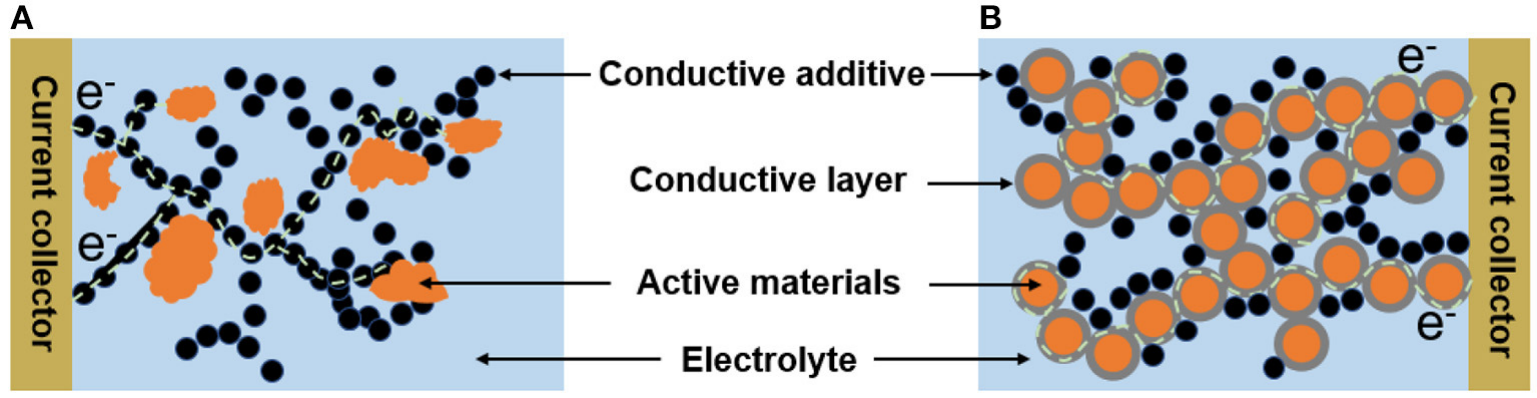
Schematic diagrams for different microstructures of the suspension electrode **(A)** “point-to-point;” **(B)** “face-to-face” contact mode between the active materials and conductive additives.

The physical properties of the suspension electrodes are mainly determined by their microstructures. In general, the conductive additives can form percolating network for electron flow and the active materials attach to the branches of the conductive additives in the suspension electrodes (Figure 1A). It has been showed that the irregular shape or larger particle size distribution of solid material will increase the viscosity of suspension electrode (Campos et al., 2013). Because the irregular shape of particles with corners and edges will catch on neighboring particles causing higher resistance to flow (Thomas, 1965). In contrast, suspensions with more uniform size distributions and greater sphericity showed lower viscosities than those with wide size distributions and anisometric particles because the fluid cannot easily enter the intergranular void spaces in solid materials or even though the fluid enters those void spaces it cannot flow or move freely within the occupied sites (Boylu et al., 2004). In addition, the content of the conductive additives should be limited to get suspension with low viscosity, but this is unfavorable for improving the electronic conductivity. Nevertheless, one should note that the using way of the conductive additives is also important. In the most suspension electrodes, the active materials and the conductive additives are “point-to-point” contact, which is not an effective way for the electron transfer between them during the charging/discharging processes (Figure 1A). In contrast, the “fact-to-face” contact obtained by coating thin conductive layer on the surface of active materials evenly can improve the electronic transfer between the active materials and conductive additives (Figure 1B). Therefore, it is possible to obtain high conductive suspension electrodes with relatively low viscosity by engineering their microstructure.

Aqueous electrochemical flow capacitors which have advantages of high power capabilities, high safety, and low cost (Presser et al., 2012), is a class of representatives of flowable electrochemical energy storage devices. These characteristics make them promising for high-rate grid applications, such as peak-shaving for renewable energy generated from solar or wind with good flexibility and scalability (Soloveichik, 2015). We have reported a new type electrochemical flow capacitors—aqueous Li-ion flow capacitor based on commercial spinel LiMn_2_O_4_ (LMO)/activated carbon (AC) system, showing high energy density and excellent cycling life (Liu et al., 2017). Nevertheless, the viscosity is relatively high and not conducive to flow.

In present work, we synthesized spherical LMO [denoted as LMO_(sph)_] and coated a thin conductive polymer—polyaniline (PANI) layer on its surface (denoted as LMO_(sph)_/PANI) because PANI has good conductivity, high theoretical specific capacitance, and environmental stability (Eftekhari et al., 2017; Liu et al., 2019). The LMO_(sph)_/PANI suspension showed low viscosity, which was about 4.5 times lower than that of irregular LMO suspension at shear rate of 100 s^−1^. In addition, the electronic conductivity of this suspension is about 10 times higher than those of spherical LMO suspension without conductive layer coating due to the contact mode between the conducting layer and the solid active material changed from “point-to-point” into “face-to-face” mode. When the LMO_(sph)_/PANI suspension matched with AC suspension to formed an aqueous Li-ion flow capacitor, it showed a record energy density of about 27.4 W h L^−1^ at a power density of 22.5 W L^−1^ under static condition to date. The flow Li-ion capacitors under intermittent flow operation were further demonstrated. This work demonstrate that high performance aqueous Li-ion flow capacitors based on suspension electrodes with low viscosity and high electronic conductivity can be obtained by the design of their microstructure, and this strategy in this work can also be extended to other flowable electrochemical energy storage systems.

## Results and Discussion

### Characterizations of LMO_(sph)_ and LMO_(sph)_/PANI Particles

For LMO_(sph)_/PANI sample, thick PANI layer can prohibit Li ions enter into LMO during the charging/discharging processes and the thickness of PANI is very important. In order to know the optimal thickness of the PANI layer, we firstly evaluated the electrochemical performance of LMO_(sph)_/PANI samples using three-electrode configuration with different mass ratio (10:1, 2:1, 1:1, 1:3, and 1:4) of LMO and aniline hydrochloride in the synthesis process. It was found that the LMO_(sph)_/PANI sample synthesized with LMO and aniline hydrochloride ratio of 2:1 exhibited the highest specific capacity (91.2 mA h g^−1^ at current density of 0.4 A g^−1^) as compared to other samples (Supplementary Figure 1). Therefore, only this sample was selected in the following study. In addition, the capacity of pure PANI is 15.85 mA h g^−1^ at a current density of 0.4 A g^−1^ and this is significantly lower than that of LMO_(sph)_ (86.61 mA h g^−1^).

**Figure 2:**
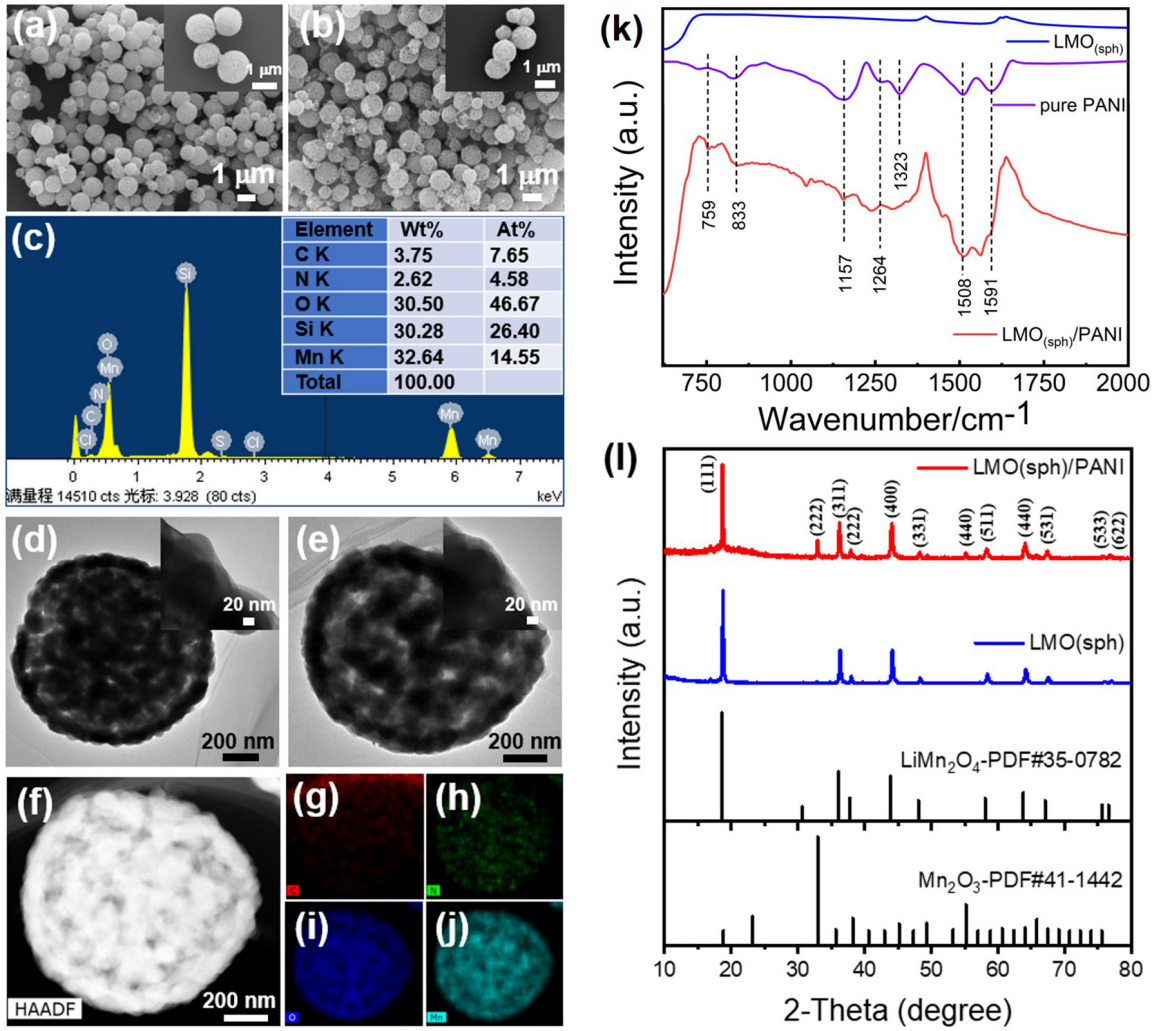
SEM images of **(a)** LMO_(sph)_ and **(b)** LMO_(sph)_/PANI, **(c)** EDX result of LMO_(sph)_/PANI. TEM images of **(d)** LMO_(sph)_ and **(e)** LMO_(sph)_/PANI. **(f)** HAADF image and EDX elemental mapping of **(g)** C, **(h)** N, **(i)** O, and **(j)** Mn elements taken from LMO_(sph)_/PANI. **(k)** FT-IR and **(l)** XRD of LMO_(sph)_, pure PANI and LMO_(sph)_/PANI.

The morphologies of the LMO_(sph)_ and LMO_(sph)_/PANI were characterized using a scanning electron microscope (SEM), and the results are shown in Figure 2a. It shows that LMO has porous spherical morphology with a rough surface and the microspheres' diameter is in the range of 0.8–1.0 μm. The morphology and size are almost unchanged after PANI coating (Figure 2b). Nevertheless, the energy dispersive spectrometer (EDS) spectrum reveals that N element can be observed after PANI coating (Figure 2c), indicating the formation of LMO_(sph)_/PANI composite (Si element comes from silicon substrate). Their morphologies and compositions were further investigated using a transmission electron microscope (TEM), and typical results are shown in Figures 2d–j. The porous LMO microsphere is the aggregate of lots of small nanoparticles, which is more clearly revealed by TEM image (Figure 2d). After PANI coating, a thin layer with thickness of about 5–20 nm can be observed on the LMO surface (Figure 2e). In addition, the elemental mapping of C, N, O, and Mn reveal that this layer is relatively uniformly coated on the surface of LMO particles (Figures 2f–j).

In order to confirm whether the surface layer was PANI or not, FT-IR was carried out, and the FT-IR spectra of LMO_(sph)_, pure PANI, and LMO_(sph)_/PANI are shown in Figure 2k. The extra peaks of LMO_(sph)_/PANI approximately located at 1591, 1508, 1264, 1157, 833, and 759 cm^−1^ are characteristic peaks of PANI. The peaks at ~1591 and ~1508 cm^−1^ are attributed to the quinoid ring stretching (N=Q=N) and benzenoid stretching (N-B-N), respectively (Li-Xiang et al., 1989; Fukuda et al., 1994; Zeng et al., 1997). The peaks at ~1264 and ~1323 cm^−1^ are assigned to the stretching vibration of C-N in the benzenoid unit, while the peak at ~1157 cm^−1^ is corresponded to the characteristic vibrational mode of the -NH^+^ (Li-Xiang et al., 1989; Fukuda et al., 1994; Zeng et al., 1997). The peak at 833 and 759 cm^−1^ are attributed to the C-H stretching. These results confirm that the coating layer is pure PANI. In addition, TGA of LMO_(sph)_/PANI composites, pure LMO(sph), and pure PANI were measured and the results are shown in Supplementary Figure 2. It reveals that the proportion of LMO and PANI is about 9:1 in the LMO/PANI composites.

The influences of PANI coating process on the crystallinity of LMO were also investigated using X-ray diffraction technique. Figure 2l shows the XRD patterns of LMO_(sph)_ and LMO_(sph)_/PANI. The pattern of LMO_(sph)_ are well-matched with spinel lithium manganate (PDF#35-0782), indicating good crystallinity and high purity for the products. Nevertheless, some new peaks corresponding to Mn_2_O_3_ (PDF#41-1442) phase can be observed after PANI coating. This indicates a slight Li^+^ dissolved out from LiMn_2_O_4_ during the process of polymerization because of the acidity of aniline hydrochloride solution (pH = 1.45).

### Characterizations and Physical Properties of LMO Suspensions

In order to better evaluate the influences of microstructure on the physical properties of LMO suspension, irregular commercial LMO particles with similar particles size distribution to that of the LMO microspheres (Supplementary Figure 3) were also used to make suspension for comparison. The weight percentages of LMO and conductive additive (ketjen black, KB) were set as 10 and 4 wt% for the suspensions. Three samples of LMO suspensions by using commercial LMO, spherical LMO without and with PANI coating were prepared, being denoted as L_(com)_10K4, L_(sph)_10K4, and L_(sph)/PANI_10K4, respectively.

**Figure 3:**
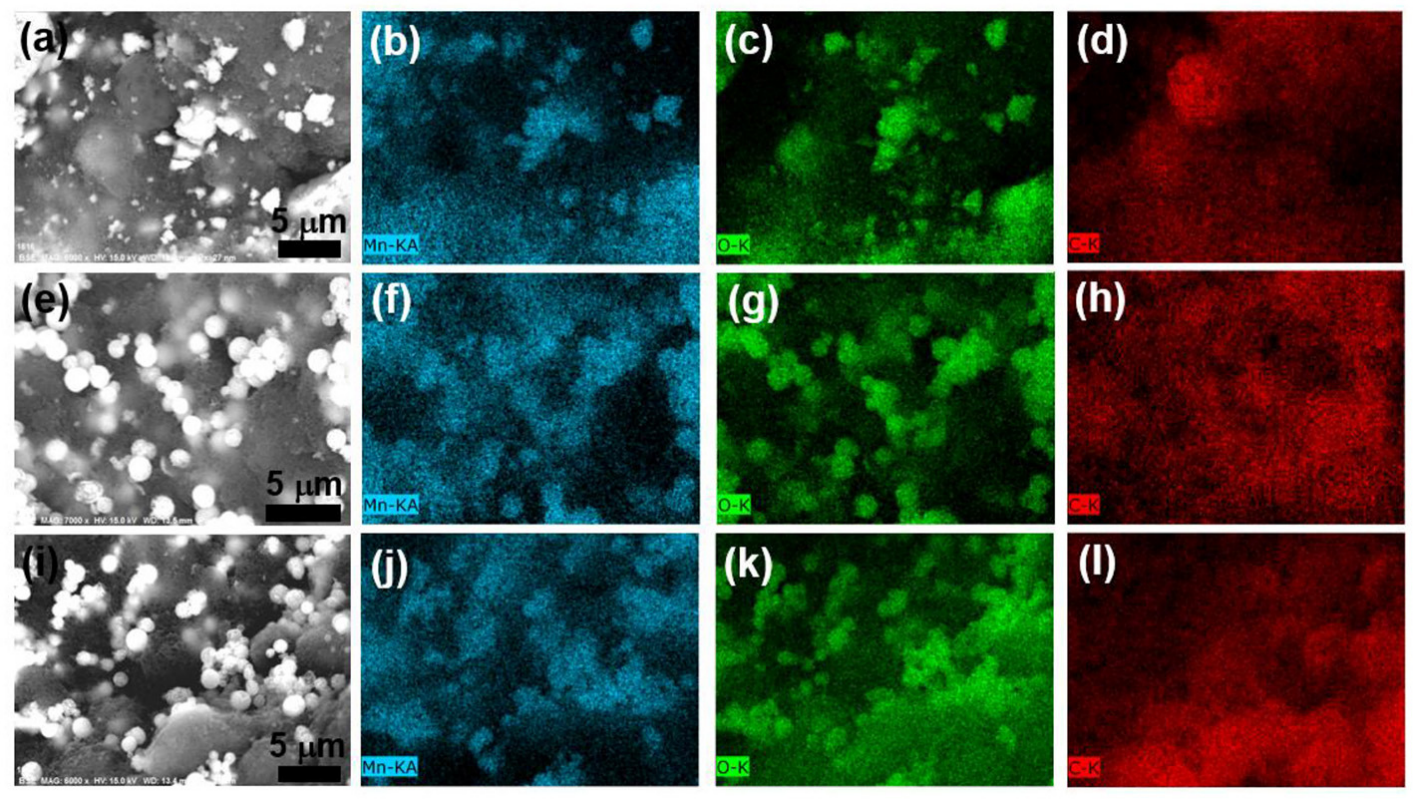
**(a,e,i)** BSE images of dry L_(com)_10K4, L_(sph)_10K4, and L_(sph)/PANI_10K4 suspensions. EDX elemental mapping of **(b,f,j)** Mn; **(c,g,k)** O and **(d,h,l)** C taken from these samples.

Figure 3 shows the morphologies and compositions of these dry suspensions using SEM. The observed high contrast regions in the backscattering electron (BSE) image in Figures 3a,e,i originate from the LMO particles. The LMO particles were uniformly dispersed in carbon matrix in all these three suspensions, as revealed by the elemental maps of Mn, O, and C (Figures 3b–d,f–h,j–l). Nevertheless, the shape of LMO particles in L_(com)_10K4 is highly irregular as compared to the other two samples.

**Figure 4:**
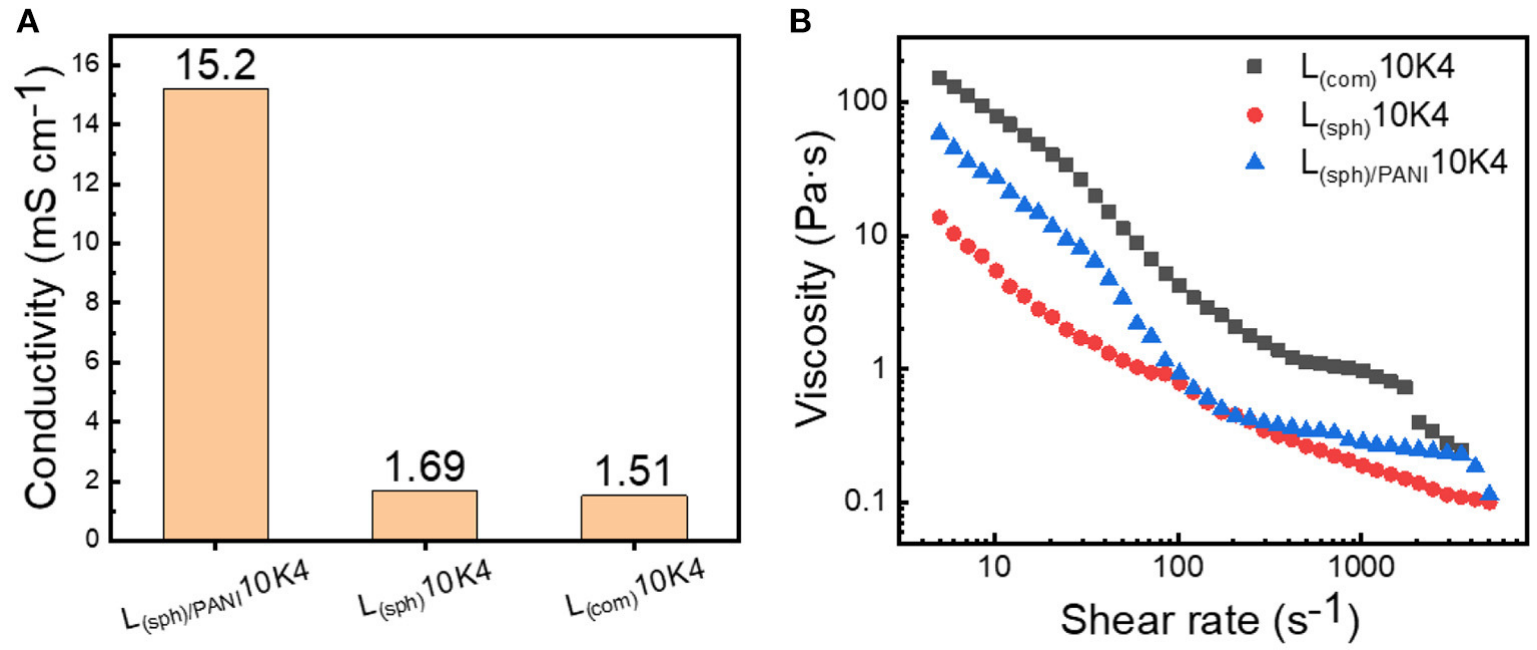
**(A)** Electronic conductivity and **(B)** viscosity curves of the L_(sph)/PANI_10K4, L_(sph)_10K4, and L_(com)_10K4 suspensions.

Figure 4A shows the electronic conductivities of these three LMO suspensions. The electronic conductivity of L_(sph)/PANI_10K4 is 15.2 mS cm^−1^, which is about 10 times higher than those of L_(com)_10K4 and L_(sph)_10K4. This indicates that PANI coating can effectively promote the electron transfer inside the suspension. In addition, the rheological behaviors of these suspensions were measured and the results are shown in Figure 4B. In general, all these suspensions exhibited strong shear-thinning behavior, i.e., the viscosity decreased with the increase of shear strain. Moreover, the viscosities of these suspensions were greatly dependent on the morphology of the solid phases. The viscosities of L_(sph)/PANI_10K4 and L_(sph)_10K4 suspensions are much lower than that of L_(com)_10K4 suspension at wide range of shear rates. For instance, the viscosities of L_(sph)/PANI_10K4 and L_(sph)_10K4 are 0.8 and 0.9 Pa·s at shear rate of 100 s^−1^, about 4.5 times lower than that of L_(com)_10K4 suspension. These results demonstrate that spherical morphology of LMO is conducive to them flow in the electrolyte because it is not easy to catch on neighboring particles with spherical shape (Thomas, 1965), resulting in lower resistance to flow and thus lower viscosity as compared to irregular morphology. In addition, we have found that LMO powders with PANI coating layer has better hydrophilic ability in the aqueous medium as compared to bare LMO powders during the preparation of the suspensions. This suggests that LMO_(sph)_/PANI has stronger interactions with water molecular in the suspension, possibly resulting in the higher viscosity of L_(sph)/PANI_10K4 as compared to that of the L_(sph)_10K4 slurry at most part of the shear rates (Singh and Pal, 2020).

### Electrochemical Properties of LMO/AC Li-Ion Flow Capacitor Tested at Static and Intermittent-Flow Condition

**Figure 5:**
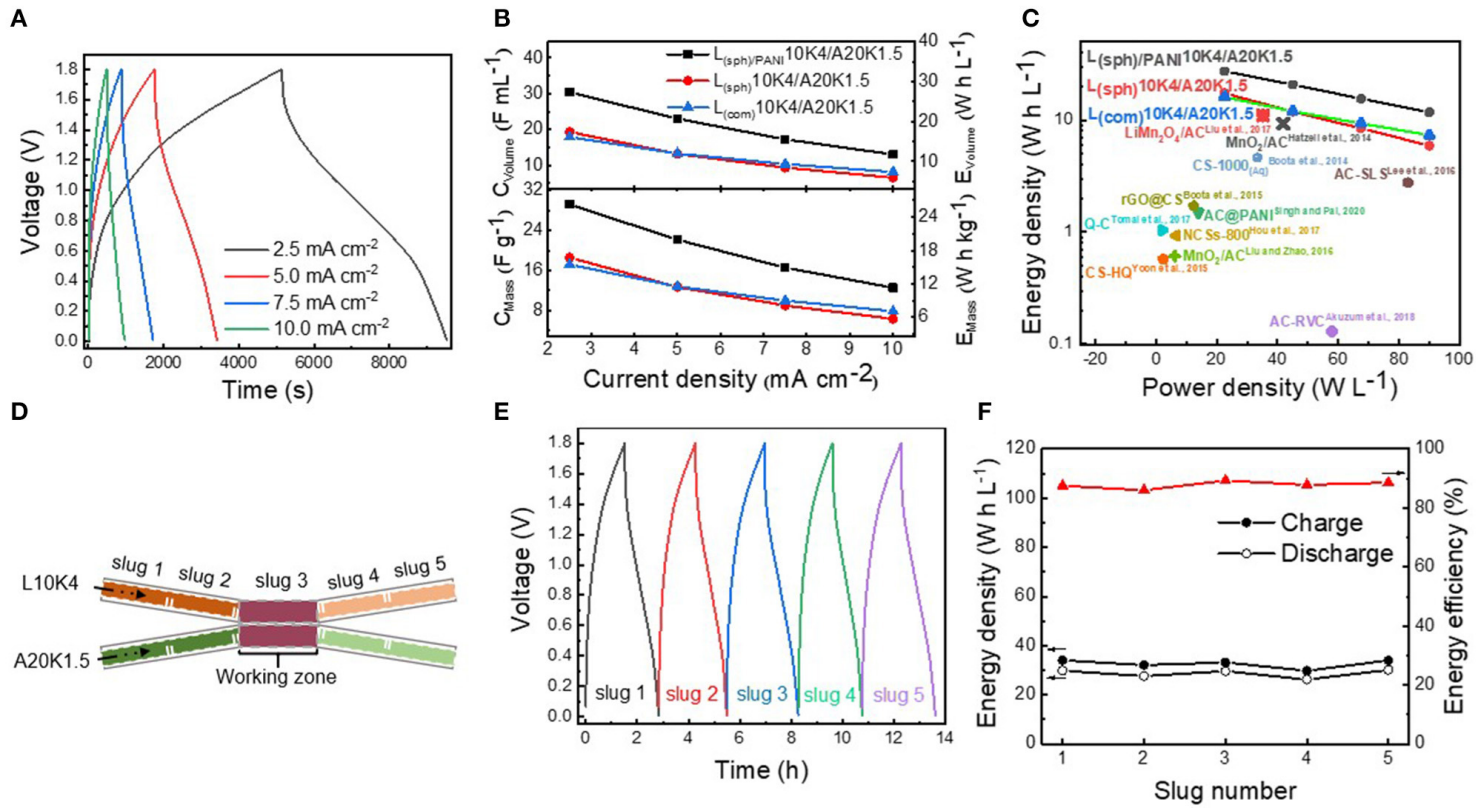
**(A)** Charging/discharging profiles of the L_(sph)/PANI_10K4/A20K1.5 static cell tested at different current densities in the voltage range of 0.0–1.8 V. **(B)** Specific capacitances (C_Mass_) and volumetric capacitances (C_Volume_) and **(C)** Ragone plot of L_(sph)/PANI_10K4, L_(sph)_10K4, and L_(com)_10K4/A20K1.5 static cell obtained at different current densities and the comparison with most other types of flow capacitors reported in literature. **(D)** Schematic of intermittent-flow tests of L10K4/A20K1.5 flow Li-ion capacitor. **(E)** Charging/discharging curves for five injections of suspensions tested at current density of 2.5 mA cm^−2^. **(F)** Energy densities and energy efficiencies of this Li-ion flow capacitor under the intermittent-flow condition.

In order to evaluate the electrochemical properties of the Li-ion flow capacitor full cell, the LMO and AC suspensions, served as the catholyte and anolyte, respectively, were matched using a static two-electrode configuration. The physical properties of AC suspension has been systemically investigated in our previous work (Liu et al., 2017), and a AC suspension consisted of 20 wt% AC and 1.5 wt% KB (denoted as A20K1.5) was used in present work. The electronic conductivity of A20K1.5 was 0.94 mS cm^−1^ and its viscosity was similar to that of L_(sph)/PANI_10K4 suspension (Supplementary Figure 4). These static cells were tested at different current densities in the voltage range of 0.0–1.8 V. Figure 5A shows the charging/discharging profiles of the L_(sph)/PANI_10K4/A20K1.5 static cell at current densities. The specific capacitances and volumetric capacitances (based on the total mass or total volume of LMO and AC suspensions) of these static cells were further calculated, and the results are shown in Figure 5B. At a current density of 2.5 mA cm^−2^, the L_(sph)/PANI_10K4/A20K1.5 static full cell delivers the highest specific capacitance of 29.2 F g^−1^ or volumetric capacitance of 30.4 F mL^−1^, respectively. In contrast, the L_(sph)_10K4/A20K1.5 and L_(com)_10K4/A20K1.5 static full cell have lower specific capacitance of 18.6 and 17.2 F g^−1^ or volumetric capacitance of 19.3 and 17.9 F mL^−1^. When the current density was increased to 10.0 mA cm^−2^, the specific and the volumetric capacitances of the L_(sph)/PANI_10K4/A20K1.5 static full cell can still be maintained at 12.5 F g^−1^ and 13.1 F mL^−1^, respectively. Meanwhile, the cycling stability of the Li-ion flow capacitor are quite stable during 500 cycles (Supplementary Figure 6). We should note that the capacity of PANI is significantly lower than that of LMO and the proportion of LMO and PANI in the LMO_(sph)_/PANI composite is about 9:1. This means that the PANI coating layer can provide some capacity in the suspension electrode, but it was mainly contributed by LMO. These results further revealed that the improvement of the electrochemical performance of the L_(sph)/PANI_10K4/A20K1.5 suspension came from the increase of electronic conductivity of the LMO_(sph)_/PANI composite. Because volumetric energy density is more meaningful than mass energy density for large scale energy storage systems, Figure 5C shows the Ragone plot of these three Li-ion flow capacitors and the volumetric energy density comparison with most other types of flow capacitors reported in literature. The L_(sph)/PANI_10K4/A20K1.5 static full cell exhibits the highest energy density and reaches to 27.4 W h L^−1^ at a power density of 22.5 W L^−1^, about 1.6 times higher than the other two static full cells at same power density. More importantly, to the best of our knowledge, this volumetric energy density is the record in aqueous electrochemical flow capacitors reported in literature, including the aqueous symmetric devices based on reduced graphene oxide-wrapped carbon spheres (rGO@CS) (Boota et al., 2015), activated carbon spheres/PANI composite (AC@PANI) (Singh and Pal, 2020), modified carbon spheres (CS-1000) (Boota et al., 2014), nitrogen-doped carbon spheres (NCSs-800) (Hou et al., 2017), activated carbon sphere coexisting with redox-active hydroquinone (CS-HQ) (Yoon et al., 2015) or quinone (Q-C) (Tomai et al., 2017), activated carbon with adding sodium lignosulfonate (AC-SLS) (Lee et al., 2016), activated carbon in the pores of reticulated vitreous carbon (AC-RVC) (Akuzum et al., 2018), and aqueous asymmetric devices such as MnO_2_/activated carbon (MnO_2_/AC) (Hatzell et al., 2014; Liu and Zhao, 2016) or LiMn_2_O_4_/AC (Liu et al., 2017) flow capacitor (Figure 5C; Supplementary Table 1). The improvement of the electrochemical performance of flow capacitor comes from not only the hybrid architecture with Li insertion-type electrode and asymmetrical configuration, but also the engineering of its microstructure. The above results clearly show the advantages of the microstructure design strategy reported in this work.

The L_(sph)/PANI_10K4/A20K1.5 full cell were also tested under intermittent-flow condition. During this step, L_(sph)/PANI_10K4 and A20K1.5 slugs were simultaneously injected into the flow cell for charging and discharging tests (Figure 5D). Figure 5E presents the charging and discharging curves for five injections of suspensions tested at a current density of 2.5 mA cm^−2^. The c harging/discharging energy densities of these five slugs are 34.1/29.8, 32.1/27.6, 33.2/29.6, 29.8/26.2, and 34.0/30.1 W h L^−1^, corresponding to energy efficiencies of 87.2, 86.4, 89.5, 88.2, and 88.0% (Figure 5F). The results demonstrate that this Li-ion flow capacitor can work smoothly under the intermittent-flow condition.

## Conclusion

In conclusion, through morphology control of solid particles in suspension, spherical LMO can effectively decrease the viscosity of suspension due to its spherical shape and uniform size (0.8–1.0 μm) which not easy to catch on neighboring particles causing lower resistance to flow. Meanwhile, PANI conductive layer coating on LMO surface can facilitate charge transfer between LMO and conductive additives because of “face-to-face” contact of them. Therefore, the L_(sph)/PANI_10K4 suspension owns high electronic conductivity of 15.2 mS cm^−1^ and low viscosity simultaneously (e.g., 0.9 Pa·s at shear rate of 100 s^−1^), much better than those of the L_(sph)_10K4 and L_(com)_10K4 suspensions. As a result, the L_(sph)/PANI_10K4/A20K1.5 Li-ion flow capacitor exhibited a record energy density of 27.4 W h L^−1^ at a power rate of 22.5 W L^−1^ under static condition. It also can work smoothly under the intermittent-flow condition, showing promise for large-scale electrochemical energy storage. In a word, the suspension electrode with high electronic conductivity and low viscosity can be realized by its microstructure design, and this strategy reported in this work can also be extended to other flowable electrochemical energy storage systems.

## Experimental Section

### Materials and Suspension Synthesis

#### Synthesis of Spherical LMO/PNAI

First, the porous LiMn_2_O_4_ microspheres were prepared using coprecipitation and high temperature calcination methods, which has been described in our previous work (Hai et al., 2019). Second, 1 g LMO and 0.5 g aniline hydrochloride powder was dispersed in 60 and 40 ml deionized water by ultrasonication and vigorous stirring, respectively. Then, the aniline hydrochloride solution was poured into LMO solution and stirred 2 h using magnetic stirrer. Third, these particles were centrifuged (8000 rpm for 3 min), and redispersed in 100 ml deionized water. 25 ml 0.4 M solution of ammonium persulphate (APS) was added into the above solution and stirred at room temperature for a period of 2 h to achieve the polymerization of aniline on the surface of LMO. Finally, the LMO/PANI composite was centrifuged and freeze-dried in vacuum.

#### Preparation of Conventional Solid Electrodes and Slurry Electrodes

The AC or LMO conventional solid electrodes were prepared by mixing 10 wt% of polyvinylidene fluoride (PVDF) binder, 10 wt% Ketjen black (ECP600JD), 80 wt% commercial LMO powder or AC (Shen Zhen Kejing Star Ltd., China), and moderate N-methyl-2-pyrrolidone (NMP) solvent and pasting on Ni foam current collector. Then the NMP solvent was removed using vacuum drying at 120°C, and pressed flat the solid electrodes under a pressure of 10 MPa cm^−2^. For fabrication of the LMO suspension electrode, the active material and conductive additive Ketjen black were weighted accurately and mixed by hand lapping. Second, the mixture and a certain amount of electrolyte were transfer into beaker and magnetic stirred for 6 h under room temperature. Then, the suspension electrode was gotten. In AC suspension, the mass ratios of AC and Ketjen black were 20 and 1.5 wt%, respectively (denoted as A20K1.5). The corresponding LMO slurries consisted of 10 wt% LMO and 4 wt% Ketjen black (denoted as L10K4).

### Characterizations

The morphologies and compositions of samples were observed using a field emission scanning electron microscopy (FESEM, SUPRA55, ZEISS, Germany) and a transmission electron microscope (TEM, FEI talos F200). X-ray diffraction (XRD) characterization was conducted to identify the crystal structure of the samples on an X-ray powder diffractometer (D8 Advance, Bruker, Germany) with a Cu Kα (λ = 0.15406 Å) radiation. Fourier Transform infrared spectroscopy spectra (FT-IR, Vertex80V, Bruker, Germany) with a resolution of 4 cm^−1^ from 400 cm^−1^ to 2000 cm^−1^. Rheological measurements were carried out on a stress-controlled rheometer (MCR72, Anton Paar) using a plate-plate geometry (plate diameter 40 mm, gap 0.5 mm) at room temperature. The shear rate was increased from 5 to 5000 s^−1^ during each test. The electronic conductivities of the LMO and AC suspensions were determined using direct current method. Supplementary Figure 5 showed the L_(com)_10K4, L_(sph)_10K4, L_(sph)/PANI_10K4, and A20K1.5 slurries' linear sweep voltammetry curve and potentiostatic polarization curve. The electronic conductivities of slurries can be obtained using the equation:

$$\begin{array}{l} \sigma =\frac{Il}{VS}, \end{array}$$

where *V* is the constant voltage of 0.25 V, *I* is the steady current after 20 min's polarization under 0.25 V, *l* is the thickness of suspension on the direction of current (*l* = 0.3 cm), and *S* is the area of suspension perpendicular to the direction of current (*S* = 1.5 cm^2^).

### Electrochemical Measurements

The electrochemical properties tests of LMO or AC conventional solid electrodes were performed using an electrochemical working station (CS 150H, Wuhan Corrtest Instrument Co., Ltd.) with three-electrode configuration, in which LMO or AC solid electrodes, Pt foil and Ag/AgCl electrode were used as working electrode, counter electrode and reference electrode, respectively. For the electrochemical tests of the suspensions, a self-made cell which consisted of carbon plate as the positive/negative electrode current collectors separated by two rubber gaskets (thickness of 1 mm) was fabricated. A groove with an active area of 20 × 5 mm^2^ as flow channel was made in each gasket. A Celgard 3,501 film was clamped by these two gaskets as separator. During electrochemical tests of full cell of the LMO/AC suspensions, the self-made cell with two channels configuration was employed at static and flow mode. In the intermittent flow mode, the slurries were pumped using a common syringe.

## Data Availability Statement

The original contributions presented in the study are included in the article/Supplementary Material, further inquiries can be directed to the corresponding authors.

## Author Contributions

DC carried out the experiment and wrote the manuscript. XB and JW participated in the experiment. HL and LL designed and supervised the experiment. HL revised the manuscript. All authors proofread the manuscript.

## Conflict of Interest

The authors declare that the research was conducted in the absence of any commercial or financial relationships that could be construed as a potential conflict of interest.
