# Supplementary material for: High Performance Aqueous Li-Ion Flow Capacitor Realized Through Microstructure Design of Suspension Electrode
Source: Front Chem. 2021 Apr 20;9:673179. doi: 10.3389/fchem.2021.673179 (PMC8093800; doi:10.3389/fchem.2021.673179)
Supplement: Supplementary file 1 [file Data_Sheet_1.PDF]

Supporting Information

High Performance Aqueous Li-Ion Flow Capacitor Realized through Microstructure Design of Suspension Electrode

Defu Cao [1], Xiaojie Bai [2], Junhui Wang [2], Hao Liu [2*], Libing Liao [1*]

[1] Beijing Key Laboratory of Materials Utilization of Nonmetallic Minerals and Solid Wastes, School of Materials Science and Technology, China University of Geosciences, Beijing 100083, China.

[2] School of Science, China University of Geosciences, Beijing 100083, China.

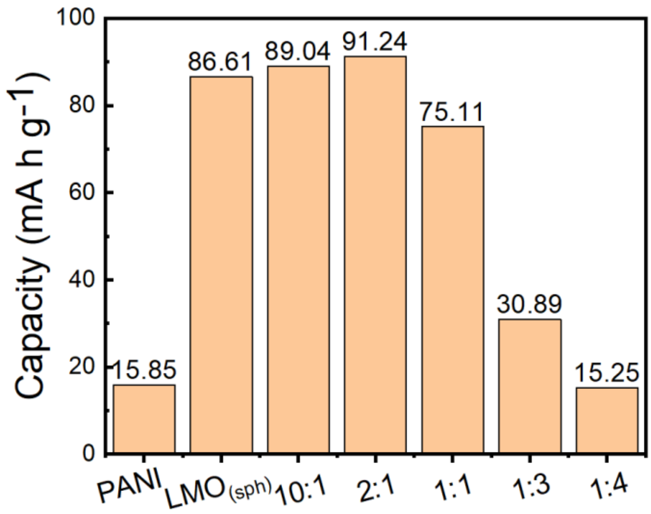

Fig S1. Capacity of PANI, $LMO_{(sph)}$ and $LMO_{(sph)}$/PANI = 10:1, 2:1, 1:1, 1:3 and 1:4 solid electrodes at a current density of 0.4 A g^{-1}(≈2.7C).

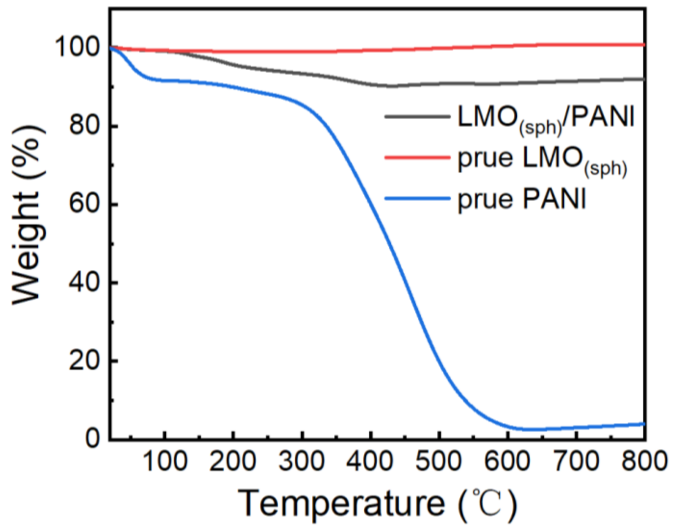

Fig S2. TGA profiles of $LMO_{(sph)}$/PANI composites, pure $LMO_{(sph)}$ and pure PANI.

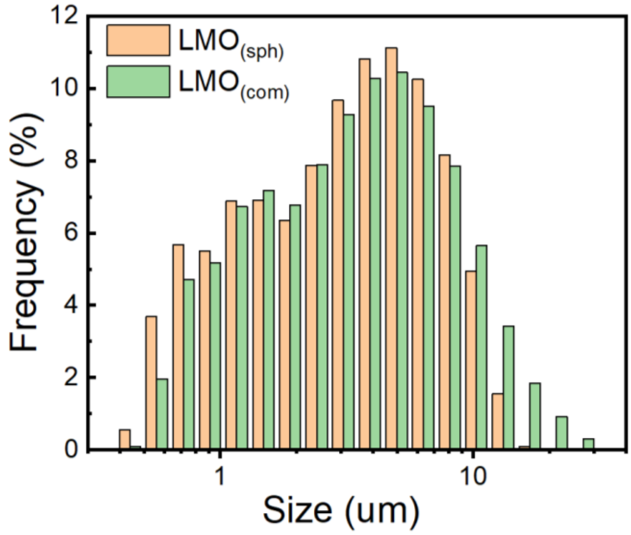

Fig S3. Laser particle size test of $LMO_{(sph)}$ and $LMO_{(com)}$ powder.

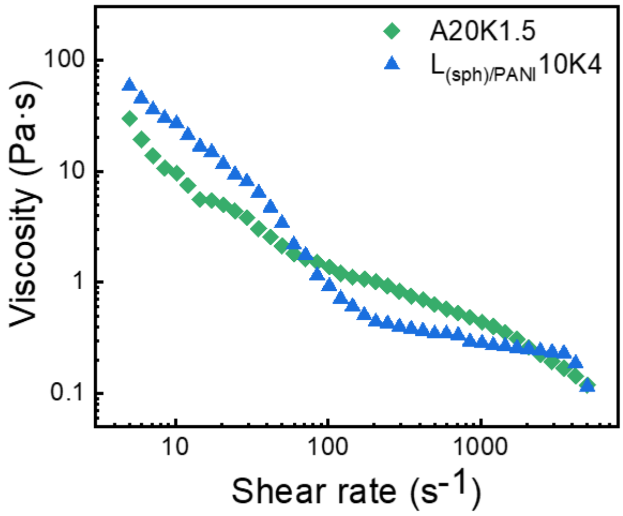

Fig S4. Viscosity curves of the $L_{(sph)/PANI}$10K4 and A20K1.5 suspensions.

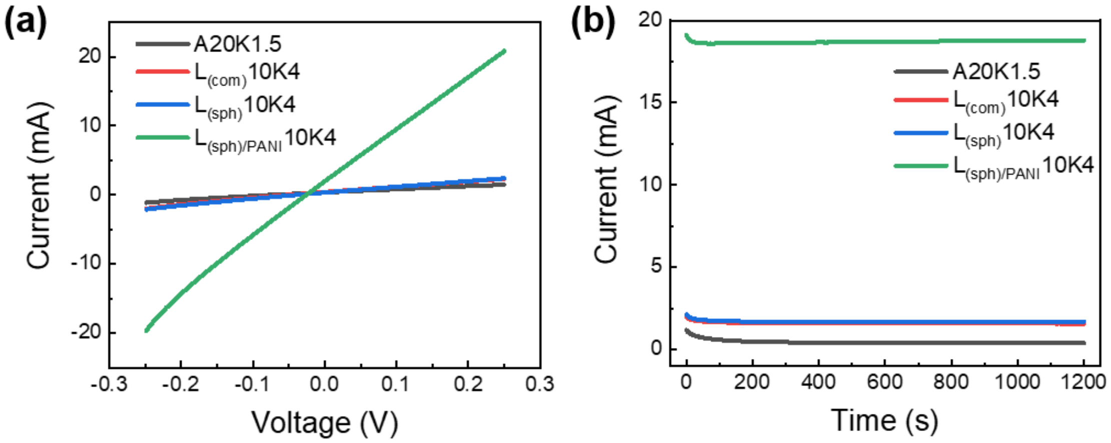

Fig S5. $L_{(com)}$10K4, $L_{(sph)}$10K4, $L_{(sph)/PANI}$10K4 and A20K1.5 slurries' (a) Linear sweep voltammetry curve (b) Potentiostatic polarization curve.

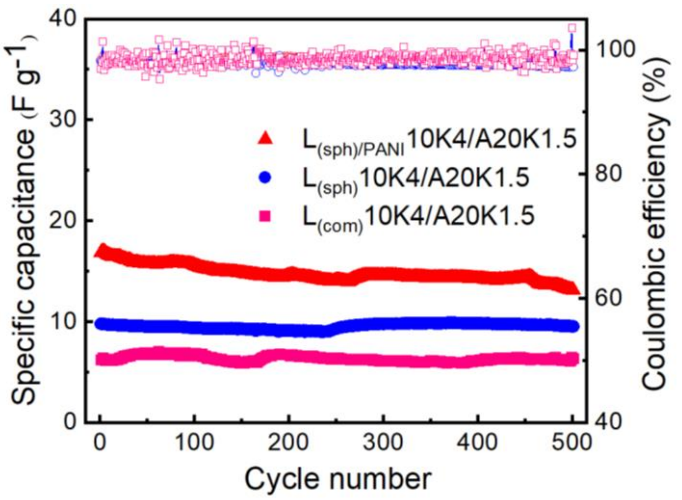

Fig S6. Long cycling performance of $L_{(sph)/PANI}$10K4, $L_{(sph)}$10K4 and $L_{(com)}$10K4/A20K1.5 static cell tested at a current density of 10.0 mA cm^{-2}.

Table S1. Comparison of volumetric energy density of aqueous electrochemical flow capacitors reported in literature.

Active materials	Voltage window	Configuration	Electrolyte	Energy density @ Power density	Ref
AC@PANI	0.8 V	Symmetric	1 M H_2SO_4	~ 1.5 W h L^{-1} @13.9 W L^{-1}	Singh and Pal, 2020
AC-RVC	1.0 V	Symmetric	1 M Na_2SO_4	~ 0.1 W h L^{-1} @58.0 W L^{-1}	Bilen Akuzum, 2018
NCSs-800	0.7 V	Symmetric	1 M H_2SO_4	~ 0.9 W h L^{-1} @6.6 W L^{-1}	Hou et al., 2017
Q-C	1.0 V	Symmetric	0.5 M H_2SO_4 + 0.05 M HCl	~1.0 W h L^{-1} @2.0 W L^{-1}	Tomai et al., 2017
AC-SLS	0.6 V	Symmetric	1 M Na_2SO_4	~ 2.8 W h L^{-1} @83.0 W L^{-1}	Lee et al., 2016
rGO@CS	1.0 V	Symmetric	1 M H_2SO_4	~ 1.7 W h L^{-1} @12.4 W L^{-1}	Boota et al., 2015
CS-HQ	1.0 V	Symmetric	1 M H_2SO_4 + 0.3 M HQ	~ 0.6 W h L^{-1} @2.3 W L^{-1}	Yoon et al., 2015
CS-1000	1.0 V	Symmetric	2 M KOH	~ 4.6 W h L^{-1} @33.4 W L^{-1}	Boota et al., 2014
MnO_2/AC	1.8 V	Asymmetric	1 M Na_2SO_4	~ 0.6 W h L^{-1} @6.1 W L^{-1}	Liu and Zhao, 2016
MnO_2/AC	1.6 V	Asymmetric	1 M Na_2SO_4	~ 9.3 W h L^{-1} @41.9 W L^{-1}	Hatzell et al., 2014
$LiMn_2O_4$/AC	1.8 V	Asymmetric	1 M Li_2SO_4	~ 11.0 W h L^{-1} @35.2 W L^{-1}	Liu et al., 2017
**$LiMn_2O_4$@ PANI/AC**	**1.8 V**	**Asymmetric**	**1 M Li_2SO_4**	**27.4 W h L^{-1} @ 22.5 W L^{-1} 15.6 W h L^{-1} @ 67.5 W L^{-1}**	**this work**

Note:

AC@PANI: The AC-PANI composite that coated polyaniline (PANI) layer on the surface of AC.

AC-RVC: A composite suspension electrode composed of AC slurry distributed in the pores of reticulated vitreous carbon (RVC).

NCSs-800: The nitrogen-doped carbon spheres (NCSs) which obtained at 800 ℃.

Q-C: A suspension electrolyte based on the impregnation of redox-active quinone derivatives in nanoporous carbon.

AC-SLS: AC suspension with 5 mM sodium lignosulfonate (SLS) added.

rGO@CS: A 3D interconnected hybrid materials composed of graphene oxide sheets (GO) and carbon spheres (CS).

CS-HQ: A suspension electrolyte based on the impregnation of 0.3 M redox-active hydroquinone (HQ) derivatives in CS.

CS-1000: A modified carbon spheres (CS) activated at 1000 ℃, and the electrolyte is aqueous.

MnO_2/AC: The positive and negative electrodes are MnO_2 and AC suspension electrodes, respectively.

$LiMn_2O_4$/AC: The positive and negative electrodes are $LiMn_2O_4$ and AC suspension electrodes, respectively.

$LiMn_2O_4$@PANI/AC: The positive and negative electrodes are $LiMn_2O_4$@PANI and AC suspension electrodes, respectively. And the $LiMn_2O_4$@PANI is $LiMn_2O_4$-PANI composite that coated polyaniline (PANI) layer on the surface of $LiMn_2O_4$.
